# varGuid: R and Python Implementations of Variance-Guided Regression for Robust Effect-Size Estimation in Linear Models

**DOI:** 10.1177/01466216261470433

**Published:** 2026-07-15

**Authors:** Sibei Liu, Zihao Wang, Min Lu

**Affiliations:** 1 Division of Biostatistics, Department of Public Health Sciences, 5452 University of Miami Miller School of Medicine, Miami, FL, USA

**Keywords:** heteroscedasticity, effect-size estimation, linear regression, analysis of variance, weighted least squares, open-source software

## Abstract

Linear regression and analysis of variance are widely used in applied psychological measurement to estimate group, condition, and covariate effects, yet statistical efficiency and conventional inference can be compromised when outcome variance changes across groups or covariate levels. This article introduces varGuid for R and varguid for Python, open-source implementations of variance-guided regression for linear models. The method estimates a covariate-dependent mean-variance relationship and uses it to iteratively reweight the original mean model. Ordinary analyses use iteratively reweighted least squares, whereas sparse analyses use an iteratively reweighted lasso. Because the original design matrix and outcome scale are retained, regression coefficients and ANOVA contrasts remain directly comparable with conventional effect estimates. Robustness here refers to relaxation of the homoscedasticity assumption rather than resistance to outliers. Under the conditions established for variance-guided regression, the estimator matches the homoscedastic baseline in population predictive quasi-risk when variance is constant and improves on that baseline when variance depends on covariates. The packages accept general linear-model design matrices, including ANOVA-style encodings, and provide baseline and variance-guided predictions, example data, and heteroscedasticity-consistent summaries for non-lasso fits. The Python implementation also supports NumPy and pandas inputs, Patsy formulas, model summaries, and a scikit-learn-compatible estimator. Both implementations are operating-system independent and require no unusual hardware. Source code, documentation, examples, and installable files are available through CRAN, PyPI, GitHub, and Zenodo. The packages provide accessible tools for applying variance-guided regression as a primary or companion analysis when homogeneity of variance is uncertain in routine measurement research and related quantitative applications.

## Description

Linear regression and analysis of variance (ANOVA) are routinely used in applied psychological measurement to estimate group, condition, and covariate effects on outcomes such as scale and test scores. When conditional variance differs across groups or covariate levels, ordinary least-squares coefficients retain their usual interpretation under standard mean-model assumptions but can be inefficient, and conventional standard errors can be misleading (White, 1980). Heteroscedasticity-consistent standard errors repair inference without changing point estimates; transformations alter the outcome scale; and conventional weighted least squares require a variance model or weights to be supplied.

The varGuid R package (Liu & Lu, 2026a) and the varGuid Python package (Wang & Lu, 2026) implement the global linear mean-variance component of variance-guided regression (Liu & Lu, 2026b). The method estimates a covariate-dependent variance relationship and iteratively reweights the original mean model, using iteratively reweighted least squares for ordinary fits or an iteratively reweighted lasso for sparse fits. Because the design matrix is retained, regression coefficients and ANOVA contrasts remain on the original outcome scale. Here, robust refers to relaxation of homoscedasticity, not resistance to outliers.

Under the assumptions of Liu and Lu (2026b), the estimator matches the homoscedastic baseline in population predictive quasi-risk when variance is constant and improves upon it when variance depends on covariates. The method can therefore be used as a primary or companion analysis without requiring analysts to establish heteroscedasticity in advance. Both packages also provide baseline and variance-guided predictions and heteroscedasticity-consistent summaries for non-lasso fits.

Both packages accept general linear-model design matrices, including ANOVA-style encodings, and provide example data, model-fitting tools, and prediction utilities. The Python implementation additionally supports NumPy and pandas inputs, Patsy formulas, model summaries, and a scikit-learn-compatible estimator.

## Supplemental Material


Supplemental Material - varGuid: R and Python Implementations of Variance-Guided Regression for Robust Effect-Size Estimation in Linear Models
Supplemental Material for varGuid: R and Python Implementations of Variance-Guided Regression for Robust Effect-Size Estimation in Linear Models by Sibei Liu, Zihao Wang, and Min Lu in Applied Psychological Measurement.

## Data Availability

varGuid 0.1.5 requires R 3.5.0 or later (R Core Team, 2026) and no compilation; varGuid 0.1.8 requires Python 3.12 or later. Both are operating-system independent with no unusual hardware requirements. Source code, documentation, examples, and installable files are available through CRAN, PyPI, GitHub, and Zenodo. The source code and documentation are publicly available through CRAN, PyPI, GitHub, and Zenodo. Supplemental files include source code, installable software, documentation, and sample input and output files. No human-participant research data are associated with this article.
