# Supplementary material for: varGuid: R and Python Implementations of Variance-Guided Regression for Robust Effect-Size Estimation in Linear Models
Source: Appl Psychol Meas. 2026 Jul 15:01466216261470433. Online ahead of print. doi: 10.1177/01466216261470433 (PMC13375855; doi:10.1177/01466216261470433)
Supplement: Supplemental Material - varGuid: R and Python Implementations of Variance-Guided Regression for Robust Effect-Size Estimation in Linear Models [file sj-zip-1-apm-10.1177_01466216261470433.zip › varGuid_CPE_Software_and_Samples/R/varGuid_manual.pdf]

Package ‘varGuid’

June 19, 2026

Type Package

Title Variance-Guided Regression Improving Upon OLS and ANOVA

Version 0.1.5

Date 2026-06-18

Author Sibei Liu [aut],
Min Lu [aut, cre]

Maintainer Min Lu <luminwin@gmail.com>

License GPL (>= 2)

Depends R (>= 3.5.0)

Imports glmnet, lmtest, sandwich

Description Fits variance-guided linear regression models that provide an alternative to ordinary least squares (OLS) for general linear-model design matrices, including ANOVA-style encodings. The methods use an iteratively reweighted least squares estimator or an iteratively reweighted lasso estimator and implement the global linear mean-variance model from the associated 2026 Statistics in Medicine article <doi:10.1002/sim.70632>. Under the assumptions in that
paper, the estimator matches the homoscedastic baseline in population predictive quasi-risk when variance is constant and improves on it when the variance depends on covariates. The grouping-based nonlinear prediction extension from Section 3 is available in the development version on GitHub.

Encoding UTF-8

LazyData true

URL https://github.com/luminwin/varGuid

BugReports https://github.com/luminwin/varGuid/issues

NeedsCompilation no

Repository CRAN

Date/Publication 2026-06-18 22:40:02 UTC

Contents

cobra2d 2
lmv 3
prd 4
varGuid 5

Index **7**

---

cobra2d *Data from the cobra2 Simulation*

Description

Data with non-linear and interaction relationship with 500 observations and 15 variables.

Usage

```
data(cobra2d)
```

Author(s)

Sibei Liu and Min Lu

References

Liu, S. and Lu, M. (2026). *Variance-Guided Regression for Heteroscedastic Data with a Grouping-Based Extension for Nonlinear Prediction. Statistics in Medicine*. 45(13-14):e70632. doi:10.1002/sim.70632

Examples

```
# library(copula)
# cobra2 = function(n = 500, d = 15, sd = .1, corrv = 0) {
#   set.seed(1)
#   d <- max(10, d)
#   X <- matrix(runif(n * d, -1, 1), ncol = d)
#   paramlist <- lapply(1:d, function(j) {list(min=-1,max=1)})
#   myCop <- normalCopula(param=rep(corrv,dim(combn(d,2))[2]), dim = d, dispstr = "un")
#   myMvd <-  mvdc(copula=myCop, margins=rep("unif",d),paramMargins=paramlist)
#   X[, 1:d] <- rMvdc(n, myMvd)
#  dta <- data.frame(list(x = X, y = X[,1]*X[,2] + X[,3]^2 - X[,4]*X[,7] + X[,8]*X[,10] - X[,6]^2
#                         + rnorm(n, sd = sd)))
#   colnames(dta)[1:d] <- paste("x", 1:d, sep = "")
#   f <- "x1 * x2 + x3 ^ 2 - x4 * x7 + x8 * x10 - x6 ^ 2"
#   fs <- "I(x1 * x2) + I(x3 ^ 2) + I(-x4 * x7) + I(x8 * x10) - I(x6 ^ 2)"
#   list(f = f, fs = fs, dta = dta)
# }

data(cobra2d)
```

lmv *Fit Variance-Guided Linear Mean-Variance Models*

Description

Fits the stage-1 variance-guided linear model for heteroscedastic data using iteratively reweighted least squares (IRLS) when `lasso = FALSE` or an iteratively reweighted lasso procedure when `lasso = TRUE`. For `lasso = FALSE`, the returned object also includes weighted least squares and heteroscedasticity-consistent inference summaries based on the final fit.

Usage

```
lmv(X, Y, M = 10, step = 1, tol = exp(-10), lasso = FALSE)
```

Arguments

`X`	Input matrix with observations in rows and predictors in columns.
`Y`	Numeric response vector.
`M`	Maximum number of iterations.
`step`	Scale parameter for the data weights.
`tol`	Tolerance parameter for convergence.
`lasso`	Logical; if `TRUE`, uses the iteratively reweighted lasso algorithm. Otherwise, IRLS is used.

Value

A list with the following components:

`beta`	Coefficient estimates from the final variance-guided fit.
`obj.OLS`	Unweighted baseline `lm` fit used to initialize IRLS when `lasso = FALSE`.
`obj.lasso`	Unweighted baseline `glmnet` fit used to initialize the iteratively reweighted lasso when `lasso = TRUE`.
`obj.varGuid`	Final fitted model from either `lm` or `glmnet`, depending on `lasso`.
`res`	Object returned by the variance-model update in the last iteration.
`obj.varGuid.coef`	For `lasso = FALSE`, a list of weighted least squares and heteroscedasticity-consistent coefficient summaries computed from the final fit.
`X`	The input design matrix `X`.

Author(s)

Sibei Liu and Min Lu

References

Liu, S. and Lu, M. (2026). *Variance-Guided Regression for Heteroscedastic Data with a Grouping-Based Extension for Nonlinear Prediction*. *Statistics in Medicine*. 45(13-14):e70632. doi:10.1002/sim.70632

Examples

```
data(cobra2d, package = "varGuid")
dat <- cobra2d
set.seed(1)
tid <- sample(seq_len(nrow(dat)), 200)
train <- dat[-tid, ]
yid <- which(colnames(dat) == "y")

o <- lmv(X = train[, -yid], Y = train[, yid], lasso = FALSE)
summary(o$obj.varGuid)
summary(o$obj.OLS)
head(prd(o, train[, -yid], model = "baseline"))
head(prd(o, train[, -yid], model = "varGuid"))

o2 <- lmv(X = train[, -yid], Y = train[, yid], lasso = TRUE)
o2$beta
o2$obj.lasso$beta
head(prd(o2, train[, -yid], model = "baseline"))
head(prd(o2, train[, -yid], model = "varGuid"))
```

---

prd *Predict from Fitted Stage-1 varGuid Models*

Description

A lightweight prediction helper for objects returned by `lmv()`. For ordinary weighted least squares fits it dispatches to `stats::predict()`. For iteratively reweighted lasso fits it dispatches to `glmnet::predict.glmnet()` and returns a numeric vector.

Usage

```
prd(object, newdata, model = c("varGuid", "baseline"), ...)
```

Arguments

`object`	An object returned by `lmv()`.
`newdata`	A matrix or data frame of predictors for prediction.
`model`	Which fitted model to use. `"varGuid"` uses the final variance-guided fit. `"baseline"` uses the initial OLS fit when `lasso = FALSE` or the initial lasso fit when `lasso = TRUE`.
`...`	Additional arguments passed to `stats::predict()` or `glmnet::predict.glmnet()`.

Details

This CRAN release covers the global linear mean-variance model from Section 2 of Liu and Lu (2026). For the grouping-based nonlinear prediction extension from Section 3 of the paper, please use the development version available at `devtools::install_github("luminwin/varGuid")`.

Value

A numeric vector of predictions.

Author(s)

Sibei Liu and Min Lu

References

Liu, S. and Lu, M. (2026). *Variance-Guided Regression for Heteroscedastic Data with a Grouping-Based Extension for Nonlinear Prediction. Statistics in Medicine*. 45(13-14):e70632. doi:10.1002/sim.70632

Examples

```
data(cobra2d, package = "varGuid")
dat <- cobra2d
set.seed(1)
tid <- sample(seq_len(nrow(dat)), 200)
train <- dat[-tid, ]
yid <- which(colnames(dat) == "y")

o <- lmv(X = train[, -yid], Y = train[, yid], lasso = FALSE)
head(prd(o, train[, -yid], model = "baseline"))
head(prd(o, train[, -yid], model = "varGuid"))

o2 <- lmv(X = train[, -yid], Y = train[, yid], lasso = TRUE)
head(prd(o2, train[, -yid], model = "baseline"))
head(prd(o2, train[, -yid], model = "varGuid"))
```

`varGuid` *Variance-Guided Regression Improving Upon OLS and ANOVA*

Description

The **varGuid** package implements the global linear mean-variance model from Liu and Lu (2026) using iteratively reweighted least squares and iteratively reweighted lasso estimation for general linear-model design matrices, including ANOVA-style encodings. This CRAN release focuses on the Section 2 linear mean-variance model and its prediction utilities for fitted stage-1 models. For the grouping-based nonlinear prediction extension from Section 3 of the paper, please use the development version available at `devtools::install_github("luminwin/varGuid")`.

Author(s)

Sibei Liu and Min Lu

References

Liu, S. and Lu, M. (2026). *Variance-Guided Regression for Heteroscedastic Data with a Grouping-Based Extension for Nonlinear Prediction*. *Statistics in Medicine*. 45(13-14):e70632. doi:10.1002/sim.70632

Examples

```
data(cobra2d, package = "varGuid")
dat <- cobra2d
set.seed(1)
tid <- sample(seq_len(nrow(dat)), 200)
train <- dat[-tid, ]
yid <- which(colnames(dat) == "y")

o <- lmv(X = train[, -yid], Y = train[, yid], lasso = FALSE)
summary(o$obj.varGuid)
summary(o$obj.OLS)
head(prd(o, train[, -yid], model = "baseline"))
head(prd(o, train[, -yid], model = "varGuid"))

# Iteratively reweighted lasso:
o2 <- lmv(X = train[, -yid], Y = train[, yid], lasso = TRUE)
o2$beta
o2$obj.lasso$beta
head(prd(o2, train[, -yid], model = "baseline"))
head(prd(o2, train[, -yid], model = "varGuid"))
```

Index

cobra2d, 2

lmv, 3

prd, 4

varGuid, 5
